# Persistent soil seed banks promote naturalisation and invasiveness in flowering plants

**DOI:** 10.1111/ele.13783

**Published:** 2021-05-24

**Authors:** Margherita Gioria, Angelino Carta, Carol C. Baskin, Wayne Dawson, Franz Essl, Holger Kreft, Jan Pergl, Mark van Kleunen, Patrick Weigelt, Marten Winter, Petr Pyšek

**Affiliations:** ^1^ Institute of Botany Department of Invasion Ecology Czech Academy of Sciences Průhonice Czech Republic; ^2^ Department of Biology, Botany Unit University of Pisa Pisa Italy; ^3^ Department of Biology University of Kentucky Lexington KY USA; ^4^ Department of Plant and Soil Sciences University of Kentucky Lexington KY USA; ^5^ Department of Biosciences Durham University Durham UK; ^6^ Centre for Invasion Biology Department of Botany and Zoology Stellenbosch University Stellenbosch South Africa; ^7^ BioInvasions Global Change, Macroecology‐Group University of Vienna Vienna Austria; ^8^ Centre for Invasion Biology Department of Botany and Zoology Stellenbosch University Stellenbosch South Africa; ^9^ Biodiversity, Macroecology and Biogeography University of Goettingen Goettingen Germany; ^10^ Ecology, Department of Biology University of Konstanz Konstanz Germany; ^11^ Zhejiang Provincial Key Laboratory of Plant Evolutionary Ecology and Conservation Taizhou University Taizhou China; ^12^ German Centre for Integrative Biodiversity Research‐iDiv, Halle‐Jena‐Leipzig Leipzig Germany; ^13^ Department of Ecology Faculty of Science Charles University Prague Czech Republic

**Keywords:** alien species, angiosperm, dormancy, exotic species, GloNAF, GloSSBank, persistence, plant invasions, seed mass

## Abstract

With globalisation facilitating the movement of plants and seeds beyond the native range, preventing potentially harmful introductions requires knowledge of what drives the successful establishment and spread of alien plants. Here, we examined global‐scale relationships between naturalisation success (incidence and extent) and invasiveness, soil seed bank properties (type and densities) and key species traits (seed mass, seed dormancy and life form) for 2350 species of angiosperms. Naturalisation and invasiveness were strongly associated with the ability to form persistent (vs. transient) seed banks but relatively weakly with seed bank densities and other traits. Our findings suggest that seed bank persistence is a trait that better captures the ability to become naturalised and invasive compared to seed traits more widely available in trait databases. Knowledge of seed persistence can contribute to our ability to predict global naturalisation and invasiveness and to identify potentially invasive flowering plants before they are introduced.

## Graphical abstract

We examine global‐scale relationships between naturalization success and invasiveness, soil seed bank properties (type and density), and key species traits (seed mass, seed dormancy, and life form) for 2350 angiosperm taxa. We found that the ability to form persistent reserves of viable seeds in the soil is a consistent indicator of the incidence and extent of naturalization and of the likelihood of them becoming invasive. We also showed that seed bank persistence is a trait that better captures the ability of angiosperms to become naturalized and invasive compared to seed traits more widely available in trait databases.

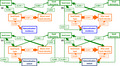

## INTRODUCTION

Understanding the drivers of successful establishment and spread of plants introduced beyond their native range of distribution is key to predicting new invasions in an era of globalisation and global environmental change (Gallien et al., 2019; Meyerson & Mooney, 2007; Pyšek et al., 2020). This is particularly important for flowering plants, given the increased risks of undetected introductions in non‐native regions associated with the rapidly increasing trade of seeds (Humair et al., 2015) and unintentional hitchhiking of seeds associated with the movement of goods and people (Anderson et al., 2015; Bradley et al., 2012). Moreover, there is evidence that new introductions may have faster rates of expansion than past or current invasions (Wilson et al., 2017), making early detection and rapid response a critical component of alien plant management (Simberloff, 2014).

Naturalised plant species are alien species that have established self‐sustaining populations outside their native distribution range. Naturalised species that have spread from the original loci of introduction are regarded as invasive (Richardson & Pyšek, 2012; Richardson et al., 2000). While much research has focused on identifying the traits that characterise invasive plants, comparatively less attention has been paid to the naturalisation stage, mainly due to difficulties in categorising naturalised and invasive occurrences and lack of comparable data (Richardson & Pyšek, 2012). However, recent efforts to integrate the increasingly available information on the naturalisation status of plant species globally (van Kleunen et al., 2015, 2019) have provided the necessary data to test hypotheses on potential mechanisms underlying naturalisation success as opposed to failure (Gallien et al., 2019; Richardson & Pyšek, 2012).

Formation of a persistent soil seed bank is one of the strategies that hedges against the risks of reproductive failure in unpredictable environments (Cohen, 1966; Venable & Brown, 1988; Childs *et al*. 2010; Venable, 2007; Larson & Funk, 2016) and may promote species persistence (Adams et al., 2005), together with other mechanisms such clonal growth, competition for light, and resprouting (Clarke et al., 2013; Grime, 2001; Honnay & Bossuyt, 2005). In a persistent soil seed bank (seed bank hereafter), dormant and/or non‐dormant seeds retain their viability for over a year or until the second germination season. In contrast, seeds in a transient seed bank remain viable for <1 year and not until the second germination season (Thompson et al., 1997; Walck et al., 2005). Reserves of viable seeds of alien species ready to germinate when conditions become favourable may be viewed as a dimension of propagule pressure (Gioria et al., 2012). Seeds may tolerate a substantially wider range of environmental conditions than living plants (Fenner & Thompson, 2005). Formation of a persistent seed bank may thus be critical to the survival of populations in non‐native areas (Gioria et al., 2012; Pyšek et al., 2015), where abiotic and biotic filters may differ from those acting in the native range (Richardson & Pyšek, 2012), especially for species regenerating exclusively from seeds. As reserves of genetic variability (Mandák et al., 2012; Templeton & Levin, 1979), persistent seed banks may affect the evolutionary potential of populations and their ability to respond to environmental variation (Donohue et al., 2005, 2010). If such an ability is superior or more rapid than that of native plants, establishment and spread of the former would be facilitated (Gioria & Pyšek, 2016). Further, seed banks may facilitate species coexistence via their storage effect (Chesson & Warner, 1981), which is associated with variation in individual‐ and species‐level responses to inter‐ and intra‐annual variation in biotic and abiotic conditions (Pake & Venable, 1996; Rees & Long, 1992). Coexistence can be beneficial to alien plants in overcoming biotic filtering in the case of functional similarities with native species or if the co‐occurring native or alien species are competitively superior (Gioria et al., 2011, 2012; Gioria & Osborne, 2014).

Although persistent seed banks are a major component of plant community dynamics (Harper, 1977), especially in early successional communities (Grime, 2001; Warr et al., 1993), hypotheses about their importance in determining naturalisation and invasiveness of alien plants have only recently been tested. Pyšek et al., (2015) found that seed bank persistence contributed to the naturalisation of 348 European species in North America indirectly, due to its positive correlation with the number of native habitats. Previous work by Gioria et al., (2019) demonstrated a positive correlation between seed bank persistence and invasiveness in 955 congeneric invasive and non‐invasive (but not necessarily naturalised) species. A lag in testing hypotheses about the importance of seed banks for invasions over large spatial scales might partly be due to the labour‐intensive and time‐consuming effort needed to collect reliable data on the persistence and dynamics of natural seed banks for many species (Thompson et al., 1997). Moreover, the persistence and accumulation potential of seeds in the soil usually have not been regarded as species traits (but see Fenner & Thompson, 2005) but as plant properties that are a *function* of certain species traits and how these traits respond to environmental variation during seed development and maturation, and after seed dispersal (Baskin & Baskin, 2014; Donohue et al., 2010; Long et al., 2015; Thompson et al., 1993, 2003). This is especially true for seed bank size (here defined as the density of seeds of a species in or on the soil), which varies largely in time and space and is affected by demographic factors (Harper, 1977) and seed predation (Hulme, 1998).

To advance our understanding of the role of seed banks in plant invasions, we evaluate how seed bank persistence relates to naturalisation and invasiveness in angiosperms, using global seed bank data collected from the native range for 2350 species. We tested two main hypotheses: (1) Naturalisation success, measured by the incidence of naturalisation (depending on whether a species has become naturalised outside its native range) and extent of naturalisation (number of regions where a species has become naturalised globally), is related to the type (persistent vs. transient) and size (density) of native soil seed banks. (2) Invasiveness (global invasive status of a species, depending on the presence or absence of invasive records outside its native distribution range) is driven by the ability of naturalised species to form persistent and/or large native seed banks. We predict that the formation of a persistent seed bank in the native range is a suitable indicator of a species’ ability to become naturalised in non‐native regions. Further, we expect that dispersal through time by a persistent seed bank promotes invasiveness by increasing the availability of windows of opportunity for successful germination over time, resulting in repeated episodes of establishment.

To test our hypotheses, we accounted for phylogenetic relatedness among the species in our database, since recent evidence has shown that seed bank persistence is phylogenetically structured in angiosperms (Gioria et al., 2020). We included seed mass, seed dormancy and species life form among the potential predictors of naturalisation and invasion success since these traits are often correlated with seed bank persistence and densities in the soil (Long et al., 2015; Moles et al., 2000; Thompson et al., 1993, 2003) as well as with naturalisation and invasiveness. Seed mass has often been found to play an important role in the invasion process (Hamilton et al., 2005; Pyšek & Richardson 2007; Pyšek *et al*. 2009; Schmidt & Drake, 2011), possibly because it is often correlated to seed dispersal ability (Howe & Smallwood, 1992), seed production (Moles *et al*. 2004) and seed persistence in the soil (Thompson et al., 1993). Small seeds and presence of dormancy are typically associated with persistent and dense seed banks compared to large, non‐dormant seeds (Fenner & Thompson, 2005; Gioria et al., 2020; Long et al., 2015). The formation of a persistent seed bank is considered more important for the persistence of annual than perennial species (Gioria et al., 2020; Thompson et al., 1998), because many of the latter use bud banks and clonal propagation for population maintenance and expansion (Salisbury 1942; Grime, 2001; Lachaise et al., 2021), while woody species are generally less likely to persist as seeds in the soil than herbaceous species (Fenner & Thompson, 2005; Gioria et al., 2020). A short life cycle is also associated with naturalisation and invasiveness (Cadotte et al., 2006; Funk et al., 2016; Pyšek et al., 2017; Schmidt & Drake, 2011). Finally, we examined potential causal relationships between seed bank properties and these species traits, and how these variables affect the incidence and extent of naturalisation and the ability of naturalised species to become invasive. Ultimately, our findings can contribute to our ability to predict invasiveness and prevent the introduction of potentially invasive plant species.

## MATERIALS AND METHODS

### Data compilation

A full description of the data and statistical procedures used to test our hypotheses is available in Appendix S1 (Supplementary Information) and a short summary is provided here. We extracted data from the Global Soil Seed Bank database (GloSSBank; Gioria et al., 2020), comprising data on viable seed banks for 2589 angiosperm taxa in 14,695 records. Each record (individual study site per species) includes information on local seed bank type (persistent vs. transient, *sensu* Thompson et al., 1997) and local seed bank density, defined as the mean number of seedlings per square metre. Based on this information and for the purpose of this paper, we created a further set of variables at the species level, by combining data at the record level: *Seed bank type* (transient vs persistent), based on whether at least one record of seed bank persistence was available. This variable provides information on the *ability* of a species to form persistent seed banks (Gioria et al., 2020). *Mean seed bank density*, defined as the arithmetic mean of local seed bank density values. *Maximum seed bank density*, defined as the maximum local seed bank density value recorded for a species. For most species (68%), multiple local seed bank density values were available. While mean seed bank density values provide an indication of how many seeds of a species are found in the soil on average, maximum seed bank density values are indicative of the potential number of seeds a species can accumulate in the soil under suitable environmental conditions.

For each species in our database, we included information on *life form* (annuals, herbaceous perennials and woody plants), based on a combination of sources or directly from the source papers (Gioria et al., 2019); *seed mass* (mg), obtained from the Royal Botanic Gardens Kew Seed Information Database (2020); and *seed dormancy* (dormant vs. non‐dormant), based on information extracted from the Baskin Dormancy Database (Baskin & Baskin, 2014; Willis et al., 2014).

Naturalisation success was defined by two variables: *Naturalisation incidence* (naturalised vs. non‐naturalised), depending on whether a species has been recorded as naturalised at least in one region globally, based on the regional classification used by van Kleunen et al., (2015); and *Naturalisation extent*, defined as number of regions where a species has been reported as naturalised globally. This information was extracted from the Global Naturalized Alien Flora (GloNAF) database (version 1.2; van Kleunen et al., 2019). *Invasiveness* was defined by the global invasion status of a species (invasive vs. non‐invasive), depending on whether a species had been reported as invasive (be it locally, regionally, or globally), and it was based on information derived directly from original papers, local and regional floras, or databases of invasive species (see Gioria et al., 2019 for details).

The final dataset used in our analyses includes seed bank data and species traits for 2350 taxa from their native range. Native records were available from a broad latitudinal range, ranging from N 78.08 to S 62.16 in latitude, including Antarctic‐ and sub‐Antarctic islands (Figure S1) and covering a broad range of ecosystems. The taxonomic status of each species was validated using The Plant List database (V.1.1, http://www.theplantlist.org/), using only those species whose taxonomic status was regarded as ‘resolved’.

### Data analysis

We used two approaches to analyse global‐scale soil seed bank data and their relationship with naturalisation and invasiveness of angiosperms (Appendix S1). To account for shared evolutionary history and avoid violating the assumption of independence among the data associated with phylogenetically relatedness (Garamszegi 2014), we performed phylogenetic generalised mixed models in a Bayesian framework (Markov Chain Monte Carlo generalised linear mixed models, MCMCglmms; Hadfield & Nakagawa, 2010), including the pruned phylogeny among the random effects. The phylogenetic tree was constructed using the R package ‘V. PhyloMaker’ (Jin & Qian, 2019), using the *bind*.*relative* function to attach taxa absent from the implemented mega‐tree by Smith and Brown (2018) to their designated genus.

We modelled, separately, three response variables (naturalisation incidence, naturalisation extent and invasiveness) as functions of three seed bank properties and three species traits (Table S1). Seed bank properties included seed bank type and seed bank density (mean or maximum, log(*x* + 1)‐transformed). Species traits included seed mass (log(*x*+1)‐transformed), seed dormancy (dormant vs. non‐dormant), and life form (annuals, perennial herbs and woody). Because seed bank density values were positively correlated with seed bank persistence, we performed three separate models for each response (nine final models are reported), including one seed bank property at a time and all three species traits (Table S2). Seed bank properties and species traits and interactions between these variables were included in these models as fixed effects. The phylogeny and species identity were used as random effects (*n* = 2350 species in models of naturalisation incidence or extent, *n* = 1253 naturalised species in models of invasiveness). These models were performed using the R package ‘MCMCglmm’ (v. 2.30; Hadfield, 2010). Binary phylogenetic models (Hadfield, 2010) were used to model, separately, naturalisation incidence (naturalised vs. non‐naturalised species) and invasiveness (invasive vs. non‐invasive species), while Gaussian phylogenetic models were used to model naturalisation extent (log(*x* + 1)‐transformed). We used weakly informative priors in all models, fixing the residual covariance matrix for binary traits while using parameter expanded priors for the random effects for continuous response variables. Each model was run for 1,000,000 MCMC steps, with an initial burn‐in phase of 10,000 and a thinning interval of 100 (de Villemereuil & Nakagawa, 2014), resulting, on average, in 9000 posterior distributions. From the resulting posterior distributions, we calculated the posterior mean, posterior mode and lambda, and 95% Highest Posterior Density (HPD) or Credible Intervals (CI). Significance of model parameters was estimated by examining CIs; parameters with CIs overlapping with zero were considered not significant.

We used structural equation modelling (SEM; Grace, 2006, 2020) to characterise the potential and assumed causal relationships between seed bank properties and species traits, and how these variables affect the incidence and extent of naturalisation, and invasiveness. Using this framework, we tested a range of hypotheses based on a priori scientific knowledge via the specifications of the corresponding models (Grace & Irvine, 2020). We used standardised coefficients as model parameter estimates, based on standard deviations of the variables in the models. Standardised coefficients can be compared directly and allow to make inferences about the relative strength of relationships between variables (Grace & Bollen, 2005). We also calculated unstandardised path coefficients, which can be used as prediction coefficient, estimating the mean influences of predictors on the response variable and the variation, as well as explanatory coefficients (Grace & Bollen, 2005). SEM models included seed bank properties (seed bank type, mean or maximum seed bank density) and species traits (seed mass, seed dormancy and life form) as drivers and naturalisation incidence, naturalisation extent, or invasiveness, with each response variable being modelled separately. All numerical variables were log(*x* + 1)‐transformed prior to analyses. Mean and maximum seed bank density were regressed on seed bank type in all models. This allowed to calculate the indirect effects of seed bank type on the response variables via seed bank density (mean or max). For model evaluation and selection, we adopted the ‘Weight of Evidence Approach’ proposed by Grace (2020), starting with considerations on sample size. We examined the maximum‐likelihood chi‐square statistic and corresponding *p* value. Assessment of model fit was based on use of multiple Approximate Fit Indices, based on recommendations by Kline (2016). SEM analyses were performed using the ‘lavaan’ R package (v. 0.6–7; Rosseel, 2012). All analyses were conducted in the R software environment (v. 4.0.3, R Development Core Team, 2020).

## RESULTS

### Naturalisation success

**FIGURE 1:**
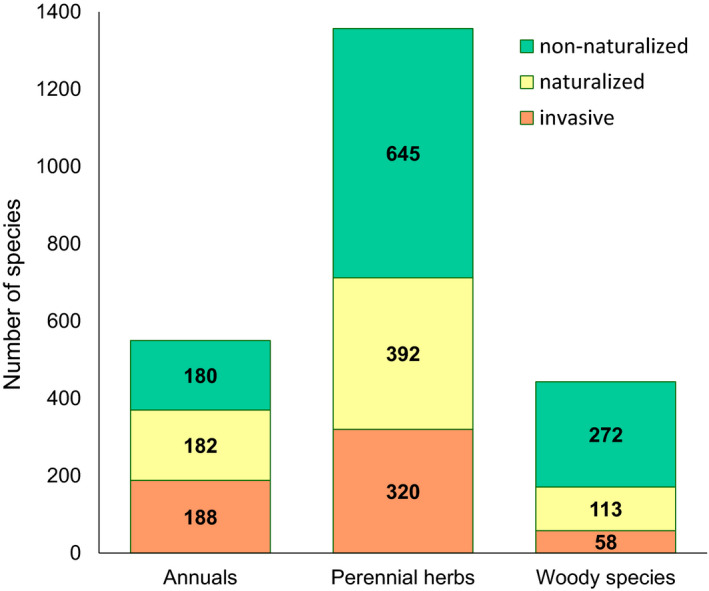
Number of species in the GloSSBank database for which seed bank records from the native range are available (*n* = 2350 species). For each life form (annuals, perennial herbs and woody species), species were classified as invasive, naturalised (but not invasive), and non‐naturalised

**FIGURE 2:**
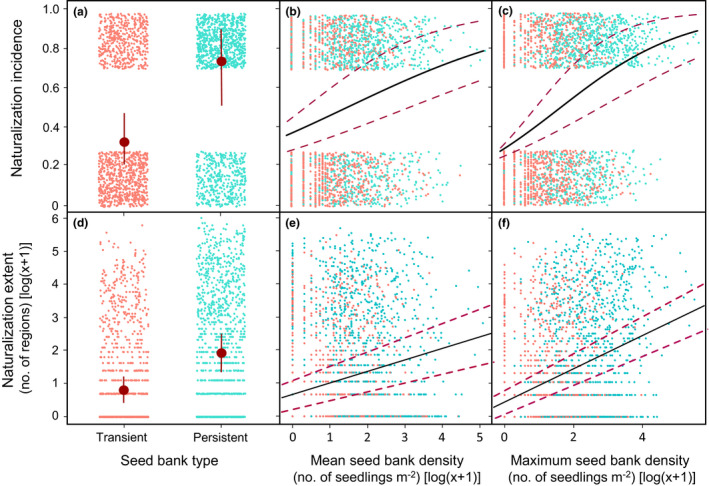
Global naturalisation incidence and extent of alien plants in relation to seed bank properties. Fitted values and credible intervals from phylogenetically informed binary models of (a–c) naturalisation incidence and (d–f) Gaussian models of naturalisation extent, in relation to (a and d) seed bank type, (b and e) mean seed bank density and (c and f) maximum seed bank density, for 2350 flowering plant species, using MCMC sampling. Persistent seed banks are displayed in turquoise, while transient seed banks are presented in salmon. Jitter points were used to display all points within each level of the binary variables: naturalisation incidence [naturalised (1) vs. non‐naturalised (0)], and seed bank type [persistent (1) vs. transient (0)]. Mean and maximum seed bank density (number of seedlings m^−2^), seed mass (mg) and naturalissation extent (number of naturalised regions) are expressed on a log(*x *+ 1)‐scale

The number of naturalised species (invasive and non‐invasive) and non‐naturalised species, by life form, is presented in Figure 1. The number of species in classes of naturalisation incidence and extent is presented in Figure S2. Table 1, Figure 2 and Table S3 report phylogenetically informed results. Species able to form persistent seed banks in the native were significantly more likely to become naturalised (*P*_MCMC_ <0.001) and did so in a significantly higher number of regions (on a log‐scale) than those forming a transient seed bank only (*P*_MCMC_ <0.001). A significant positive association was also found between both naturalisation incidence and extent with mean (*P*_MCMC_ <0.001) or maximum (*P*_MCMC_ <0.001) seed bank densities. Annual herbs were significantly more likely to become naturalised than perennial herbs (*P*_MCMC_ <0.001), while woody species were significantly less likely to become naturalised than herbaceous species, in all models of naturalisation incidence. Annual species have also become naturalised in a significantly greater number of regions compared to perennial herbs and woody species (*P*_MCMC_ <0.001). None of the species’ traits (seed dormancy, seed mass and life form) and none of their interactions with seed bank type and mean or maximum seed bank density had a significant effect on the probability of a species to become naturalised or the extent of naturalisation.

**TABLE 1:** Results of species‐level generalised mixed models with Bayesian estimation (MCMCglmms), modelling naturalisation incidence (1–3) and naturalisation extent (4–6), for 2350 flowering plant taxa and invasiveness (7–9) for 1253 flowering plant taxa, based on seed bank data from the native range

Model	Posterior mean	Lower 95% C.I.	Upper 95% C.I.	*p*_MCMC_
Naturalisation models				
1. Naturalisation incidence (*n* = 2350)				
Seed bank type (Persistent)	1.217	0.591	2.114	<0.001
Life form (Annual)	1.088	0.307	2.11	<0.001
Life form (Woody)	−1.076	−2.276	−0.202	0.005
2. Naturalisation incidence (*n* = 2350)				
Mean seed bank density [log(*x* + 1)]	0.748	0.332	1.48	<0.001
Life form (Annual)	1.064	0.321	2.245	<0.001
Life form (Woody)	−1.296	−1.297	−0.304	<0.001
3. Naturalisation incidence (*n* = 2350)				
Maximum seed bank density [log(*x* + 1)]	1.006	0.617	1.766	<0.001
Life form (Annual)	0.913	0.278	1.605	<0.001
Life form (Woody)	−1.006	−1.974	−0.268	0.004
4. Naturalisation extent (*n* = 2350)				
Seed bank type (Persistent)	0.962	0.813	1.085	<0.001
Life form (Annual)	0.518	0.349	0.68	<0.001
5. Naturalisation extent (*n* = 2350)				
Mean seed bank density [log(*x* + 1)]	0.152	0.241	0.374	<0.001
Life form (Annual)	0.538	0.316	0.702	<0.001
6. Naturalisation extent (*n* = 2350)				
Maximum seed bank density [log(*x* + 1)]	0.214	0.186	0.24	<0.001
Life form (Annual)	0.511	0.341	0.679	<0.001
Invasion models				
7. Invasiveness (*n* = 1253)				
Seed bank type (Persistent)	0.923	0.117	2.39	0.004
8. Invasiveness (*n* = 1253)				
Mean seed bank density [log(*x* + 1)]	0.889	0.043	2.052	0.002
9. Invasiveness (*n* = 1253)				
Maximum seed bank density [log(*x* + 1)]	2.003	0.36	6.035	<0.001

Note

Posterior mean values and credible intervals (CI) are presented. Only explanatory variable exerting significant effects are presented in the models. Reconstructed phylogeny and species identity were included in the models as random factors (Table S1).

**FIGURE 3:**
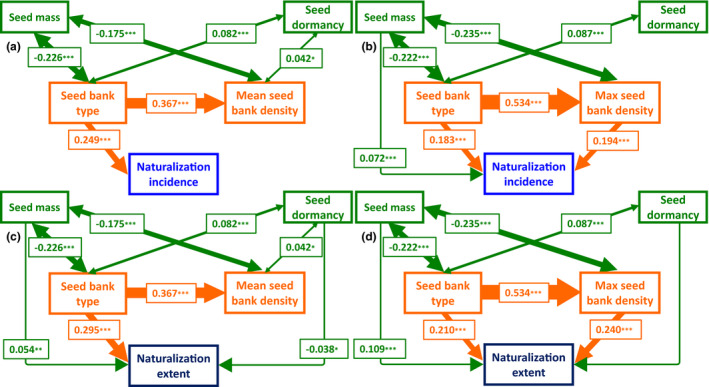
Structural equation model considering all plausible pathways of direct and indirect effects on the naturalisation incidence (a and b) and naturalisation extent (c and d), including direct effects of seed bank properties (type, mean density and maximum density) and seed traits (seed mass and seed dormancy) and indirect effect of seed bank type naturalisation incidence and extent through seed bank density (mean and max density), for 2350 flowering plant species, based on seed bank data from the native range. Seed bank density is described by (a,c) mean density values or (b and d) maximum density values, at the species level. Only significant standardised coefficients are presented (*p ≤ 0.05; **p ≤ 0.01; ***p ≤ 0.001, *z* test). The thickness of the solid and dashed arrows reflects the magnitude of the standardised SEM coefficients. Mean and maximum seed bank density (number of seedlings m^−2^), seed mass (mg), and naturalisation extent (number of naturalised regions) are expressed on a log(*x *+ 1)‐scale. Fit indices of the tested structural equation models and *R* ^2^ values are presented in Table S5

Structural equation models (SEM) showed that seed bank type was the only variable among the predictors (seed bank properties and species traits) consistently having significant and relatively strong direct effects on naturalisation incidence and extent (Figure 3 and Table S4). Models including life form showed a poorer fit compared to those excluding life form (these results are not presented). Models excluding life form represented a good fit based on the fit statistics and cut‐offs commonly recommended to evaluate structural equation models (Table S5; Kline, 2016). Seed bank type had a significant direct effect on both naturalisation incidence and extent. Seed bank type also exerted a significant indirect effect (IE) on naturalisation incidence (IE_nat_incidence_ =0.104, estimate =0.103, *P_z_* <0.001) and extent (IE_nat_extent_ =0.128, estimate =0.192, *P_z_* <0.001), through its positive and significant effect on maximum seed bank density. Seed dormancy and mass had only weak or non‐significant effects on naturalisation incidence and extent, consistent with the findings of phylogenetic models. In all models assessing naturalisation incidence and extent, seed mass was negatively correlated with seed bank type and density (mean or maximum), indicating that small, but not large, seeds are correlated with persistent and dense seed banks. On the contrary, seed dormancy was positively correlated with seed bank persistence and mean seed bank density, but not with maximum seed bank density.

### Invasiveness of naturalised plants

Naturalised species able to form persistent seed banks were significantly more likely to become invasive than those forming transient seed banks only (*P*_MCMC_ =0.004) (Table 1 and S1 and Figure S3). Naturalised species with higher mean seed bank density (*P*_MCMC_ =0.002) or maximum seed bank density (*P*_MCMC_ <0.001) were also more likely to become naturalised. None of the species’ traits (seed dormancy, seed mass and life form) and none of their interactions with seed bank type and mean or maximum seed bank density had significant effects on the probability of naturalised species to become invasive.

SEM models showed that seed bank type had a significant direct effect on the invasiveness of naturalised species as well as a significant indirect effect via maximum seed bank density (IE_invasion_ =0.094, estimate =0.096, *P_z_* <0.001) (Figure S4 and Table S4). Models including life form among the potential predictors of invasiveness showed a poor fit compared to those without life form; thus, this variable was excluded from the models. Seed mass and dormancy did not have significant direct effects on invasiveness. Correlations between seed traits and seed bank properties were similar to those found in models of naturalisation incidence. Persistent seed banks were correlated significantly with small seed mass and dormant seeds, while seed bank density was negatively correlated with seed mass but not significantly with dormancy. SEMs of invasiveness, however, provided a lower fit compared to models of naturalisation incidence and extent, as shown by the lower Comparative Fit Index values and higher Root Mean Square Error of Approximation and Standardized Root Mean Square Residual (Table S5). This result was possibly due to the lower number of species on which these models were tested (*n* = 1253) compared to models of naturalisation incidence (*n* = 2350).

## DISCUSSION

Our results, based on the largest soil seed bank dataset compiled so far and correcting for phylogenetic relatedness, show that a species’ ability to form persistent seed banks in the native range is a good indicator of its naturalisation and invasion potential. Species able to form persistent seed banks were twice more likely to become naturalised and did so in a greater number of regions than species forming only transient ones. Naturalised species forming persistent seed banks were also more likely to become invasive than those forming transient seed banks. Seed mass and seed dormancy were, in contrast, only weakly associated with either naturalisation success (incidence and extent) or invasiveness. The positive effects of the ability to form persistent seeds on the incidence and extent of naturalisation were evident not only for short‐lived species, which depend heavily on the ability to disperse through time for survival (Gremer & Venable, 2014; Thompson et al., 1998), but also for perennial herbs. The latter represented the majority of species in the dataset (58% vs. 23% of annuals). A greater percentage of annuals in our study have become naturalised (67%) compared to perennial herbs (52%) and woody species (39%). This is consistent with the representation of life histories in the Global Naturalized Alien Flora database (Pyšek et al., 2017) and supports evidence of the importance of persistent seed banks in the survival and expansion of annual plants (Adams et al., 2005; Gremer & Venable, 2014; Harper, 1977).

A higher probability of naturalised species to become invasive found for species able to form persistent rather than only transient seed banks suggests that seed persistence in the soil increases recruitment opportunities occurring over time, facilitating the establishment of new populations as well as their spread. In grassland ecosystems and for many alien plants, especially annual grasses, these windows of opportunity for germination and seedling establishment coincide with periods when competition for resources with native species is low, potentially promoting establishment and expansion even in alien species that are competitively inferior to natives (Gioria & Pyšek, 2017; Gioria et al., 2018). In this respect, rapid evolutionary changes towards seed survival in seed banks or optimisation of the timing of germination are demographic adaptations that may facilitate range expansions in alien plants (Blossey et al., 2017), especially in cases of intense competitive interactions between alien and native species (Gioria et al., 2019; Gioria & Osborne, 2014). Persistent seed banks may be especially important for alien species characterised by short‐distance dispersal and those that rely exclusively on seed for reproduction (Gioria et al., 2012). A positive association with invasiveness might also reflect the importance of persistent seed banks in the establishment and spread of plant populations in disturbed habitats (Harper, 1977; Thompson et al., 1998; Warr et al., 1993), where invasive plants can be highly successful (D'Antonio et al., 1999; Davis et al., 2000; Hierro et al., 2006).

Naturalisation and invasiveness were also positively associated with mean and maximum seed bank densities in models accounting for phylogenetic relatedness. This partly reflects the fact that persistent seed banks tend to be denser than transient seed banks (Gioria et al., 2020). Structural equation models, however, showed significant direct effects seed bank type on naturalisation incidence and extent as well as on invasiveness via its positive effects on maximum seed densities in the soil but not mean densities. While confirming the importance of accounting for phylogeny in large scale seed bank studies (Gioria et al., 2020), our findings suggest that the high spatio‐temporal variability of seed densities and their dependence on local environmental conditions and population characteristics (Fenner & Thompson, 2005; Harper, 1977; Thompson et al., 1997) makes this variable a relatively weak predictor of naturalisation or invasiveness. Yet, knowledge of seed densities in the soil and their persistence is key the effective management of alien plants and the restoration of native communities (Gioria et al., 2012).

The use of seed bank data collected from the native range only enabled us to circumvent the lack of information, for many studies, on differences in residence time, that is time since introduction of an alien species in a community or a region (Pyšek et al., 2015). Residence time might strongly influence the size of local seed banks in the alien range, by affecting the accumulation potential of seeds in the soil associated with multiple seed‐rain events as well as the demographic structure and densities of alien plant populations (Gioria et al., 2019). Moreover, dominance of alien plants in the standing vegetation often increases with residence time, with positive feedbacks being observed between population densities above‐ground and seed densities in the soil (Gioria & Pyšek, 2016; Robertson & Hickman, 2012). Using seed bank data from the native range also allowed us to avoid a further complication associated with observed phenotypic or rapid adaptive responses of certain seed traits to the new conditions encountered in the introduced range (Hierro et al., 2005, 2020; Maron et al., 2004). These traits include, for some invasive plants, increased seed production (Correia et al., 2016), greater seed mass in the alien range (Buckley et al., 2003; Hierro et al., 2020; Pichancourt & van Klinken, 2012), differences in the depth of seed dormancy (Kudoh et al., 2007; Udo *et al*. 2017) or in seed germination percentages and rates (Gioria & Pyšek, 2017), or alterations in the proportion of dormant versus non‐dormant seeds (Alexander & D’Antonio, 2003).

A stronger effect of seed bank type on naturalisation success and invasiveness than seed traits, found in our study, might partly reflect the fact that seed dormancy and seed mass are not consistent predictors of the ability of plants to disperse through time (Gioria et al., 2020; Long et al., 2015; Thompson et al., 1993, 2003). Seed persistence in the soil is not restricted to species with seed dormancy (Fenner & Thompson, 2005; Gioria et al., 2020; Harper, 1977; Thompson et al., 2003). Moreover, non‐dormant seeds may fail to germinate in the absence of suitable conditions for germination and some may remain viable in the soil for many years (Baskin & Baskin, 1985). Examinations of the relationship between seed mass and seed persistence have also provided contrasting results, with evidence of both negative (Bakker et al., 1996; Bekker et al., 1998; Thompson et al., 1993, 1998) and positive correlations (Leishman & Westoby, 1998; Moles et al., 2000; Moles & Westoby, 2006).

Weak or non‐significant effects of seed mass and seed dormancy are consistent with evidence that factors such as residence time, propagule pressure, climatic suitability, native range size and number of native habitats tend to play a greater role than biological traits in predicting naturalisation or invasiveness (Hamilton et al., 2005; Pyšek & Richardson 2007; Pyšek *et al*. 2009, 2020; Gallagher *et al*. 2015; Feng et al., 2016). Seed mass can contribute to naturalisation and invasiveness in opposing ways, with greater naturalisation success for larger‐seeded species, and greater invasiveness in smaller‐seeded species (Pyšek et al. 2009; Moodley et al., 2013). Such a contribution may vary in relation to the spatial scale of the study (local/habitat, regional, or continental) and across life forms (herbs vs. woody) (Hamilton et al., 2005; Pyšek & Richardson 2007; Pyšek *et al*. 2009; Gallagher *et al*. 2015) and differ depending on whether phylogenetic relatedness is accounted for (Lavoie et al., 2016). In our study, models accounting for phylogeny showed a non‐significant contribution of seed mass and dormancy as determinants of naturalisation success and invasiveness, as opposed to structural equation models, which showed a weak effect of these traits on naturalisation extent. The fact that the spread of alien species is often facilitated by human‐mediated long‐distance dispersal might mask the relative importance of seed mass and other seed traits in the invasion process (Gioria et al., 2019).

In conclusion, we showed that the ability to form persistent reserves of viable seeds in the soil is a consistent indicator of the incidence and extent of naturalisation and of the likelihood of them becoming invasive. Our findings also suggest that seed persistence in the soil is a plant property that better captures the ability of flowering plants to become naturalised and spread compared to seed traits that are more widely available in trait databases, such as seed mass and seed dormancy (Moles *et al*. 2004; Larson & Funk, 2016). Clearly, formation of a persistent seed bank is only one of the factors to be considered when attempting to predict naturalisation and invasion success and is only part of the complex strategy promoting species persistence in a community. Yet, our findings support the idea that seed bank persistence can be interpreted as a species trait (Fenner & Thompson, 2005; Gioria et al., 2020) that should be considered to prevent the introduction of potentially invasive plant species and to prioritise control of alien plants before they form substantial reserves of persistent seeds. A key challenge is to understand how seed persistence in the soil interacts with biotic and abiotic filters in promoting naturalisation and invasiveness and how it may affect the distribution of naturalised and invasive plants. Future lines of research include the collection of seed persistence data on a large scale, using multiple, alternative approaches, such as laboratory‐controlled ageing (Long *et al*. 2008) or long‐term burial experiments (Skálová et al., 2019). They also include broadening our understanding of how a persistent seed bank might affect a species’ ability to respond to climatic and other environmental changes, which could further promote establishment and expansion of naturalised and invasive populations.

## AUTHOR CONTRIBUTIONS

MG and PP conceived the idea. MG, PP and AC designed methodology. MG compiled the global soil seed bank database. WD, FE, HK, JP, MvK, PW, MW and PP compiled the Global Naturalized Alien Flora database. MG and AC analysed the data. MG wrote the manuscript with inputs from all authors.

## Supporting information

Supplementary Material

## Data Availability

Data for this article, including species names, soil seed bank data (seed bank type and mean seed bank density) from the native distribution range, species traits data (seed dormancy, seed mass and life form), and data sources are available from the Dryad Digital Repository (https://doi.org/10.5061/dryad.8sf7m0cjh; Gioria et al., 2020). Data on naturalisation incidence and naturalisation extent are extracted from the GloNAF database (van Kleunen et al. 2019). Data on naturalisation incidence and naturalisation extent, global invasion status and maximum seed bank density, for 2350 flowering plant species are presented as supporting information.
