# Supplementary material for: Persistent soil seed banks promote naturalisation and invasiveness in flowering plants
Source: Ecol Lett. 2021 May 24;24(8):1655–67. doi: 10.1111/ele.13783 (PMC8361993; doi:10.1111/ele.13783)
Supplement: Supplementary file 1 — Supplementary Material [file ELE-24-1655-s001.docx]

**Supporting Information**

**Appendix S1 Material and Methods**

**Data compilation**

To test hypotheses on whether naturalization and invasiveness of flowering plants are related to the ability to form persistent soil seed banks and accumulate large number of seeds in the soil, we extracted data from the GloSSBank (Global Soil Seed Bank) database (Gioria et al. 2020). This database comprises data for 2,589 angiosperm taxa in 862 genera and 140 families, extracted from 195 source papers reporting data on viable seed banks. Each record in the database corresponds to information on the seed bank properties of individual species at individual study sites (records), for a total of 14,695 records. Since we were interested only in the viable component of the seed bank, we included seed bank data estimated by the seedling emergence approach (Thompson et al. 1997) and seed bank estimates based on the sieving and counting of large seeds whose viability and germinability had been tested. We excluded estimates based on seed extraction methods, since they may overestimate the potential number of seeds that could germinate under suitable conditions for dormancy break and germination if viability tests are not performed. Moreover, Reinhardt & Leon (2018) showed that seed extraction vs. germination methods provide dramatically different quantitative data on seed banks in weed communities, and that the two methods are not strongly correlated, limiting the possibility of generating a conversion factor between methods. Further, the seedling emergence method is the most widely used approach to estimate seed banks (Mahé et al. 2021) and records based on this approach comprised over 97% of the records in an expanded version of our database (which includes seed bank assessments based on other methods). Our choice allowed us to avoid any potential confounding effect of the seed bank estimation method that could mask the real contribution of seed bank properties to the naturalization and invasion of alien plants. Further, we focused on seed bank persistence (and density) data collected from natural seed banks, while we excluded estimates based on the use of other approaches, such as seed burial experiments or laboratory-controlled aging (Long et al. 2008), to minimize potential noise in the data.

Each record in GloSSBank includes information on two seed bank variables recorded at individual study sites: local seed bank type (persistent vs. transient), and local seed bank density, defined as the mean number of seedlings per square meter (Gioria et al. 2020). Based on this information and for the purpose of this paper, we created a further set of seed bank variables, by combining data at the record level to derive information at the species level: *Seed bank type*, based on whether at least one record of seed bank persistence was available; *Mean seed bank density*, defined as the mean of local seed bank density values; and *Maximum seed bank density*, defined as the maximum local seed bank density value recorded for a species. While local seed bank type reflects the effect of local environmental conditions on species traits affecting seed persistence in the soil, species seed bank type provides information on the *ability* of a species to form persistent seed banks (Gioria et al. 2020). For most species (68%), multiple local seed bank density values were available. For the remaining species, mean and maximum seed bank density values coincide. While mean seed bank density values provide an indication of how many seeds of a species are found in the soil on average, across multiple sites and habitat types, maximum seed bank density values are indicative of how many seeds a species can accumulate in the soil under suitable environmental conditions. For this paper, calculations of species-level seed bank data were based only on record-level data from the native range.

Since some life history traits are known to affect seed persistence in the soil (Long et al. 2015), for each species in our database we included information on *life form* (annuals, herbaceous perennials, and woody plants), based on a combination of sources or directly from the source papers (Gioria et al. 2019); *seed mass* (mg), obtained from the Royal Botanic Gardens Kew Seed Information Database (2020); and *seed dormancy* (dormant vs. non-dormant). Information on seed dormancy was extracted from the Baskin Dormancy Database (Baskin & Baskin 2014; Willis et al. 2014), with species being classified as dormant if they had physical, physiological, morphological dormancy or a combination thereof after dispersal.

To evaluate relationships between seed bank properties and the naturalization and invasive potential of flowering plants, we also included biogeographical information. Naturalization success was defined by two variables: *Naturalization incidence* (naturalized vs. non-naturalized), depending on whether a species has been recorded as naturalized at least in one region globally, based on the regional classification used by van Kleunen et al. (2015); and *Naturalization extent*, defined as the number of regions where a species has been reported as naturalized globally. This information was extracted from the Global Naturalized Alien Flora (GloNAF) database (version 1.2; van Kleunen et al. 2019). *Invasiveness* was defined by the global invasion status (invasive vs. non-invasive) of a species, depending on whether a species had been reported as invasive (be it locally, regionally, or globally), and it was based on information derived directly from the original papers, local and regional floras, or databases of invasive species (see Gioria et al. 2019 for details). For each record, we extracted information on the local origin status of each species (alien vs. native), depending on whether a species was native or alien at the study site; this information was derived directly from the source papers or extracted from regional or local floras (Gioria et al. 2019).

For the purpose of this study, we extracted seed bank data at the species level only using records from the native range (see Discussion). The final dataset used in this study comprises information for 2,350 species, based on data extracted from 11,893 records. Native records were available from a broad latitudinal range, with records ranging from N 78.08 to S 62.16 in latitude, including Antarctic- and subantarctic islands (Fig. S1). Overall, a broad range of systems is covered, including those characterized by high level of stress, such as deserts, mountains, and Arctic/Antarctic regions. The taxonomic status of each species was validated using The Plant List database (V.1.1, http://www.theplantlist.org/). The final seed bank dataset includes only those species whose taxonomic status was regarded as ‘resolved’.

**Data analysis**

We used two approaches to analyze global-scale soil seed bank data and their relationship with naturalization and invasiveness of angiosperms. To account for shared evolutionary history and avoid violating the assumption of independence among the data associated with phylogenetic relatedness (Garamszegi 2014), we performed phylogenetic generalized mixed models in a Bayesian framework (Markov Chain Monte Carlo generalized linear mixed models, MCMCglmms; Hadfield & Nakagawa 2010), including a pruned phylogeny among the random effects. The phylogenetic tree was constructed using the R package ‘V.PhyloMaker’ (Jin & Qian 2019), using the *bind.relative* function to attach taxa absent from the implemented mega-tree by Smith & Brown (2018) to their designated genus. We modelled three response variables, separately, i.e., naturalization incidence, naturalization extent (log(*x*+1)-transformed), and invasiveness, as functions of three seed bank properties and three species traits. Seed bank properties included seed bank type, mean seed bank density (log(*x*+1)-transformed), and maximum seed bank density ((log(*x*+1)-transformed). Species traits included seed mass (log(*x*+1)-transformed), seed dormancy (dormant vs non-dormant), and life form (annuals, perennial herbs, and woody species). Because seed bank densities were positively correlated with seed bank persistence, we performed three separate models for each response, including only one seed bank property at the time and all three species traits in all models. Seed bank properties and species traits and interactions between these variables were included in these models as fixed effects (Table S1). The phylogeny (effects due to the phylogenetic differences of the species in the database) and species identity (effects due to nonphylogenetic differences between the species in the database) were used as random effects (*n* = 2,350 species in models of naturalization incidence or extent, *n* = 1,253 naturalized species (721 of which being classified as invasive) in models of invasiveness). Models of naturalization incidence or extent were based on data for 2,350 species, while models of global invasion status were based on data for 1,253 naturalized species, of which 721 were classified as invasive. These models were performed using the R package ‘MCMCglmm’ (v. 2.30; Hadfield 2010). Binary phylogenetic models (Hadfield 2010) were used to model, separately, two binary response variables: naturalization incidence (naturalized vs. non-naturalized species) and invasiveness (invasive vs. non-invasive species). Gaussian phylogenetic models were used to model naturalization extent (log(*x*+1)-transformed). We used weakly informative priors in all models, fixing the residual covariance matrix for binary traits while using parameter expanded priors for the random effects for continuous response variables (see R code below). Each model was run for 1,000,000 MCMC steps, with an initial burn-in phase of 10,000 and a thinning interval of 100 (de Villemereuil & Nakagawa 2014), resulting, on average, in 9,000 posterior distributions. From the resulting posterior distributions, we calculated mean parameter estimates and 95% Highest Posterior Density (HPD) and Credible Intervals (CI). Posterior mean is a precision weighted average of the prior mean and data mean. Significance of model parameters was estimated by examining CIs where parameters with CIs overlapping with zero were considered not significant.

Second, we used structural equation modeling (SEM; Grace 2006, 2020) to characterize the potential and assumed causal relationships between seed bank properties and species traits, and how these variables affect the incidence and extent of naturalization, and invasiveness. We used standardized coefficients as model parameter estimates, based on standard deviations of the variables in the models. Within each model, the magnitude of standardized coefficients can be compared directly to make inferences about the relative strength of relationships between variables (Grace 2005). Using this framework, we tested a range of hypotheses based on a priori scientific knowledge via the specification of the corresponding models (Grace & Irvine 2020). SEM models included seed bank properties (seed bank type and mean seed bank density, or seed bank type and maximum seed bank density) and species traits (seed mass, seed dormancy, and life form) as drivers and naturalization incidence, naturalization extent, or invasiveness, with each response variable being modeled separately. Mean and maximum seed bank density were regressed on seed bank type in all models. This allowed to calculate the indirect effects of seed bank type on the response variables via seed bank density (mean or max). For model evaluation and selection, we adopted the ‘Weight of Evidence Approach’ proposed by Grace (2020), starting with considerations on sample size (*n* = 2,350 species to test hypotheses on naturalization incidence and extent, and *n* = 1,253 to test hypotheses on global invasion status). We examined the maximum‐likelihood chi‐square statistic and corresponding *p*-value. Assessment of model fit was based on the use of multiple Approximate Fit Indices, based on recommendations by Kline (2016). SEM analyses were performed using the ‘lavaan’ R package (v. 0.6-7; Rosseel 2012). All analyses were conducted in the R software environment (v. 4.0.3, R Development Core Team 2020). A list of all variables used in phylogenetic models and structural equation modeling is provided in Table S1. A description of the response variables and fixed effects used in phylogenetic models is presented in Table S2.

R code for the priors, using the package MCMCglmm:

1. Models of naturalization incidence:

prior1 <- list(R = list(V = 1, n = 0, fix = 1), G = list(G1 = list(V = 1, n = 2), G2 = list(V = 1, n = 2)))

1. Models of naturalization extent:

prior2 <- list(R=list(V=1, nu=0.2), G=list(G1=list(V=1, nu=0.2, alpha.mu=0, alpha.V=1e3), G2=list(V=1, nu=0.2, alpha.mu=0, alpha.V=1e3)))

1. Models of invasiveness:

prior3 <- list(R = list(V = 1, n = 0, fix = 1), G = list(G1 = list(V = 1, n = 2), G2 = list(V = 1, n = 2)))

**References Appendix 1**

Baskin, J.M. & Baskin, C.C. (1985). Does seed dormancy play a role in the germination ecology of *Rumex crispus*? Weed Sci., 33, 340-343.

Bollen, K.A. (1989). *Structural equations with latent variables*. John Wiley & Sons, New York.

de Villemereuil, P. & Nakagawa, S. (2014). General quantitative genetic methods for comparative biology. In *Modern phylogenetic comparative methods and their application in evolutionary biology* (ed Garamszegi, L. Z.). Springer-Verlag, Berlin, pp. 287−303.

Gioria, M., Le Roux, J.J., Hirsch, H., Moravcová, L. & Pyšek, P. (2019). Characteristics of the soil seed bank of invasive and non-invasive plants in their native and alien distribution range. *Biol. Invasions*, 21, 2313–2332

Gioria, M., Pyšek, P., Baskin, C.C., Carta, A. (2020). Phylogenetic relatedness mediates persistence and density of soil seed banks. *J. Ecol*., 108, 2121−2131.

Grace, J.B. & Bollen, K.A. (2005). Interpreting the results from multiple regression and structural equation models. *Bull. Ecol. Soc. Am*., 86, 283−295.

Grace, J.B. & Irvine, K.M. (2020). Scientist’s guide to developing explanatory statistical models using causal analysis principles. *Ecology*, 101, e02962. https://doi.org/10.1002/ecy.2962.

Grace, J.B. (2020). A 'Weight of Evidence' approach to evaluating structural equation models. *One Ecosystem*, 5, e50452. doi:10.3897/oneeco.5.e50452.

Grace, J.B. (2006). *Structural equation modeling and natural systems*. Cambridge University Press, Cambridge.

Hadfield, J.D. & Nakagawa, S. (2010). General quantitative genetic methods for comparative biology: phylogenies taxonomies and multi-trait models for continuous and categorical characters. *J.* *Evolution. Biol.*, 23, 494−508.

Hadfield, J.D. (2010). MCMC methods for multi-response generalized linear mixed models: The MCMCglmm R package. *J. Stat. Softw.*, 33, 1−22.

Long, R.L. et al. (2008). Seed persistence in the field may be predicted by laboratory-controlled aging. *Weed Sci*., 56, 523–528.

Long, R.L. et al. (2015). The ecophysiology of seed persistence: a mechanistic view of the journey to germination or demise. *Biol. Rev. Camb. Philos. Soc*., 90, 31−59.

Mahé, I. et al. (2021). Soil seedbank: Old methods for new challenges in agroecology? *Ann. Appl. Biol.*, 178, 23–38.

Jin, Y. & Qian, H. (2019). V.PhyloMaker: an R package that can generate very large phylogenies for vascular plants. *Ecography*, 42, 1353−1359.

Kline, R.B. (2016). *Principles and practice of structural equation modeling*. 4th ed. Guilford Press, New York.

R Development Core Team. (2020). *R: a language and environment for statistical computing*. R Foundation for Statistical Computing, Vienna.

Reinhardt, T. & Leon, R.G. (2018). Extractable and germinable seedbank methods provide different quantifications of weed communities. *Weed Sci*., 66, 715–720.

Rosseel, Y. (2012). lavaan: An R Package for Structural Equation Modeling. *J. Stat. Softw*., 48, 1−36. Available at: http://www.jstatsoft.org/v48/i02/.

Royal Botanic Gardens Kew. (2020). Seed Information Database (SID). Version 7.1. Available at: http://data.kew.org/sid/.

Smith, S.A. & Brown, J.W. (2018). Constructing a broadly inclusive seed plant phylogeny. *Am. J. Bot*., 105, 302–314.

Thompson, K., Bakker, J.P. & Bekker, R.M. (1997). *Soil seed banks of NW Europe: Methodology, density and longevity*. Cambridge University Press, Cambridge.

van Kleunen, M. et al. (2015). Global exchange and accumulation of non-native plants. *Nature*, 525, 100−103.

van Kleunen, M. et al. (2019). The Global Naturalized Alien Flora (GloNAF) database. *Ecology*, 100, e02542. https://doi.org/10.1002/ecy.2542.

Willis, C.G. et al. (2014). The evolution of seed dormancy: environmental cues, evolutionary hubs, and diversification of the seed plants. *New Phytol*., 203, 300−309.

**Table S1** List of species-level variables used in phylogenetic models and structural equation models.

| **Variables** | **Type of variable** | **Variable description** |
| --- | --- | --- |
| **Naturalization incidence** | binary | 0 = not naturalized |
|  |  | 1 = naturalized in at least one world regions (GloNAF) |
| **Naturalization extent** | continuous | number of world regions where a species has become naturalized (GloNAF) |
| **Invasiveness** | binary | 0 = not invasive globally |
|  |  | 1 = invasive in at least one world region |
| **Seed bank properties** |  |  |
| Species seed bank type | binary | 0 = transient (seed viability < 1 year) |
|  |  | 1 = persistent (seeds viability > 1 year) |
| Mean seed bank density | continuous | Mean seed bank density value for each species |
| Maximum seed bank density | continuous | Maximum seed bank density value for each species |
|  |  |  |
| **Species traits** |  |  |
| Seed dormancy | binary | 0 = nondormant |
|  |  | 1 = dormant |
| Seed mass | continuous | seed mass (mg) (Kew Seed Information Database, 2020) |
| Life form | categorical | annual herb, perennial herb, woody |

**Table S2** List of phylogenetic analyses performed in this study.

| **Hp** |  | **Response variables** | **Model type** | **Fixed effects** | ***n*** |
| --- | --- | --- | --- | --- | --- |
| Hp 1a |  | Naturalization incidence | Binary MCMCglmms | species seed bank type, seed dormancy, life form, and interactions among these variables | 2,350 |
|  |  | Naturalization incidence | Binary MCMCglmms | mean seed bank density, seed dormancy, life form, and interactions among these variables | 2,350 |
|  |  | Naturalization incidence | Binary MCMCglmms | maximum seed bank density, seed dormancy, life form, and interactions among these variables | 2,350 |
| Hp 1b |  | Naturalization extent | Gaussian MCMCglmms | species seed bank type, seed dormancy, life form, and interactions among these variables | 2,350 |
|  |  | Naturalization extent | Gaussian MCMCglmms | mean seed bank density, seed dormancy, life form, and interactions among these variables | 2,350 |
|  |  | Naturalization extent | Gaussian MCMCglmms | maximum seed bank density, seed dormancy, life form, and interactions among these variables | 2,350 |
| Hp 2 |  | Invasiveness | Binary MCMCglmms | species seed bank type, seed dormancy, life form, and interactions among these variables | 1,253 |
|  |  | Invasiveness | Gaussian MCMCglmms | mean seed bank density, seed dormancy, life form, and interactions among these variables | 1,253 |
|  |  | Invasiveness | Gaussian MCMCglmms | maximum seed bank density, seed dormancy, life form, and interactions among these variables | 1,253 |

**Table S3** Results of species-level generalized mixed models with Bayesian estimation (MCMCglmms), modelling (a) naturalization incidence and (b) naturalization extent (*n* = 2,350 flowering plant taxa), and (c) invasiveness (*n* = 1,253 flowering plant taxa), based on seed bank data from the native range. Posterior mean values and credible intervals (C.I.) are presented. Only explanatory variable exerting significant effects are presented in the models.

|  | **model** |  | **posterior mean** | **lower 95% C.I.** | **upper 95% C.I.** | ***p*_MCMC_** | **phylogeny** | **species** | **units** |
| --- | --- | --- | --- | --- | --- | --- | --- | --- | --- |
| **Naturalization models** | |  |  |  |  |  |  |  |  |
|  | 1. Naturalization incidence (*n* = 2,350) |  |  |  |  |  |  |  |  |
|  | Seed bank type (Persistent) |  | 1.217 | 0.591 | 2.114 | <0.001 | 7.347 (1.063 – 19.439) | 10.441 (0.146 – 32.869) | 1.000 (1.000 - 1.000) |
|  | Life form (Annual) |  | 1.088 | 0.307 | 2.110 | <0.001 | 7.347 (1.063 – 19.439) | 10.441 (0.146 – 32.869) | 1.000 (1.000 - 1.000) |
|  | Life form (Woody) |  | -1.076 | -2.276 | -0.202 | 0.005 | 7.347 (1.063 – 19.439) | 10.441 (0.146 – 32.869) | 1.000 (1.000 - 1.000) |
|  | 2. Naturalization incidence (*n* = 2,350) | |  |  |  |  |  |  |  |
|  | Mean seed bank density [log(x+1)] |  | 0.748 | 0.332 | 1.480 | <0.001 | 9.715 (1.130 - 29.872) | 10.518 (0.109 – 38.025) | 1.000 (1.000 - 1.000) |
|  | Life form (Annual) |  | 1.064 | 0.321 | 2.245 | <0.001 | 9.715 (1.130 - 29.872) | 10.518 (0.109 – 38.025) | 1.000 (1.000 - 1.000) |
|  | Life form (Woody) |  | -1.296 | -1.297 | -0.304 | <0.001 | 9.715 (1.130 - 29.872) | 10.518 (0.109 – 38.025) | 1.000 (1.000 - 1.000) |
|  | 3. Naturalization incidence (*n* = 2,350) | |  |  |  |  |  |  |  |
|  | Maximum seed bank density [log(x+1)] |  | 1.006 | 0.617 | 1.766 | <0.001 | 5.486 (0.952 – 15.204) | 3.992 (0.122 – 18.902) | 1.000 (1.000 - 1.000) |
|  | Life form (Annual) |  | 0.913 | 0.278 | 1.605 | <0.001 | 5.486 (0.952 – 15.204) | 3.992 (0.122 – 18.902) | 1.000 (1.000 - 1.000) |
|  | Life form (Woody) |  | -1.006 | -1.974 | -0.268 | 0.004 | 5.486 (0.952 – 15.204) | 3.992 (0.122 – 18.902) | 1.000 (1.000 - 1.000) |
|  | 4. Naturalization extent (*n* = 2,350) |  |  |  |  |  |  |  |  |
|  | Seed bank type (Persistent) |  | 0.962 | 0.813 | 1.085 | <0.001 | 0.679 (0.395 – 1.010) | 1.390 (0 – 2.212) | 0.864 (0.031 - 2.206) |
|  | Life form (Annual) |  | 0.518 | 0.349 | 0.680 | <0.001 | 0.679 (0.395 – 1.010) | 1.390 (0 – 2.212) | 0.864 (0.031 - 2.206) |
|  | 5. Naturalization extent (*n* = 2,350) | |  |  |  |  |  |  |  |
|  | Mean seed bank density [log(x+1)] |  | 0.152 | 0.241 | 0.374 | <0.001 | 0.813 (0.453 - 1.323) | 1.309 (0.000 - 2.393) | 1.309 (0.000 - 2.319) |
|  | Life form (Annual) |  | 0.538 | 0.316 | 0.702 | <0.001 | 0.813 (0.453 - 1.323) | 1.309 (0.000 - 2.393) | 1.309 (0.000 - 2.319) |
|  | 6. Naturalization extent (*n* = 2,350) | |  |  |  |  |  |  |  |
|  | Maximum seed bank density [log(x+1)] |  | 0.214 | 0.186 | 0.240 | <0.001 | 0.865 (0.506- 1.268) | 1.112 (0.000 - 2.097) | 1.054 (0.035 - 2.161) |
|  | Life form (Annual) |  | 0.511 | 0.341 | 0.679 | <0.001 | 0.865 (0.506- 1.268) | 1.403 (0.000 - 2.097) | 1.054 (0.035 - 2.161) |
| **Invasion models** | |  |  |  |  |  |  |  |  |
|  | 7. Invasiveness (*n* = 1,253) |  |  |  |  |  |  |  |  |
|  | Seed bank type (Persistent) |  | 0.923 | 0.117 | 2.390 | 0.004 | 2.136 (0.151 – 6.543) | 14.338 (0.127 – 80.837) | 1.000 (1.000 - 1.000) |
|  | 8. Invasiveness (*n* = 1,253) | |  |  |  |  |  |  |  |
|  | Mean seed bank density [log(x+1)] |  | 0.889 | 0.043 | 2.052 | 0.002 | 2 (0.140 - 5.852) | 10.731 (0.133 – 55.196) | 1.000 (1.000 - 1.000) |
|  | 9. Invasiveness (*n* = 1,253) | |  |  |  |  |  |  |  |
|  | Maximum seed bank density [log(x+1)] |  | 2.003 | 0.360 | 6.035 | <0.001 | 6.003 (0.175 – 26.274) | 43.042 (0.116 – 251.383) | 1.000 (1.000 - 1.000) |

**Table S4** Results of structural equation models, showing significant direct effects and correlations among explanatory variables. Both standardized and unstandardized (estimate, in brackets) coefficients are reported. Mean and maximum seed bank density (no. of seedlings m^-2^), seed mass (mg), and naturalization extent (no. of naturalized regions) are expressed on a log(*x*+1)-scale.

| **Direct effects** |  |  | **Estimate** | **Standardized coefficient** | ***P*(>\|*z*\|)** |  | **Covariances** |  |  | **Estimate** | **Standardized coefficient** | ***P*(>\|*z*\|)** |
| --- | --- | --- | --- | --- | --- | --- | --- | --- | --- | --- | --- | --- |
| **Model 1** |  |  |  |  |  |  |  |  |  |  |  |  |
| Mean seed bank density [log] | ~ | seed bank type | 0.676 | 0.367 | <0.001 |  | seed bank type | ~~ | seed mass [log] | -0.051 | -0.226 | <0.001 |
| Naturalization incidence | ~ | seed bank type | 0.248 | 0.249 | <0.001 |  |  | ~~ | seed dormancy | 0.012 | 0.082 | <0.001 |
|  |  |  |  |  |  |  | mean seed bank density [log] | ~~ | seed mass [log] | -0.067 | -0.175 | <0.001 |
|  |  |  |  |  |  |  |  | ~~ | seed dormancy | 0.011 | 0.042 | 0.037 |
| **Model 2** |  |  |  |  |  |  |  |  |  |  |  |  |
| Max seed bank density [log] | ~ | seed bank type | 1.136 | 0.534 | <0.001 |  | seed bank type | ~~ | seed mass [log] | -0.05 | -0.222 | <0.001 |
| Naturalization incidence | ~ | seed bank type | 0.182 | 0.183 | <0.001 |  |  | ~~ | seed dormancy | 0.013 | 0.087 | <0.001 |
|  | ~ | max seed bank density [log] | 0.091 | 0.194 | <0.001 |  | max seed bank density [log] | ~~ | seed mass [log] | -0.095 | -0.235 | <0.001 |
|  | ~ | seed mass [log] | 0.08 | 0.072 | <0.001 |  |  |  |  |  |  |  |
| **Model 3** |  |  |  |  |  |  |  |  |  |  |  |  |
| Mean seed bank density [log] | ~ | seed bank type | 0.676 | 0.367 | <0.001 |  | seed bank type | ~~ | seed mass [log] | -0.051 | -0.226 | <0.001 |
| Naturalization extent [log] | ~ | seed bank type | 0.442 | 0.295 | <0.001 |  |  | ~~ | seed dormancy | 0.012 | 0.082 | <0.001 |
|  | ~ | seed mass [log] | 0.09 | 0.054 | 0.008 |  | mean seed bank density [log] | ~~ | seed mass [log] | -0.067 | -0.175 | <0.001 |
|  |  |  |  |  |  |  |  | ~~ | seed dormancy | 0.011 | 0.042 | 0.037 |
| **Model 4** |  |  |  |  |  |  |  |  |  |  |  |  |
| Max seed bank density [log] | ~ | seed bank type | 1.136 | 0.534 | <0.001 |  | seed bank type | ~~ | seed mass [log] | -0.05 | -0.222 | <0.001 |
| Naturalization extent [log] | ~ | seed bank type | 0.315 | 0.21 | <0.001 |  |  | ~~ | seed dormancy | 0.013 | 0.087 | <0.001 |
|  | ~ | max seed bank density [log] | 0.169 | 0.24 | <0.001 |  | max seed bank density [log] | ~~ | seed mass [log] | -0.095 | -0.235 | <0.001 |
|  | ~ | seed mass [log] | 0.182 | 0.109 | <0.001 |  |  |  |  |  |  |  |
|  | ~ | seed dormancy | -0.122 | -0.049 | 0.01 |  |  |  |  |  |  |  |
| **Model 5** |  |  |  |  |  |  |  |  |  |  |  |  |
| Mean seed bank density [log] | ~ | seed bank type | 0.645 | 0.326 | <0.001 |  | seed bank type | ~~ | seed mass [log] | -0.055 | -0.268 | <0.001 |
| Invasiveness | ~ | seed bank type | 0.245 | 0.238 | <0.001 |  |  | ~~ | seed dormancy | 0.015 | 0.099 | <0.001 |
|  |  |  |  |  |  |  | mean seed bank density [log] | ~~ | seed mass [log] | -0.059 | -0.153 | <0.001 |
|  |  |  |  |  |  |  |  | ~~ | seed dormancy | 0.016 | 0.057 | 0.042 |
| **Model 6** |  |  |  |  |  |  |  |  |  |  |  |  |
| Max seed bank density [log] | ~ | seed bank type | 1.217 | 0.541 | <0.001 |  | seed bank type | ~~ | seed mass [log] | -0.056 | -0.271 | <0.001 |
| Invasiveness | ~ | seed bank type | 0.12 | 0.118 | <0.001 |  |  | ~~ | seed dormancy (D) | 0.014 | 0.097 | <0.001 |
|  | ~ | max seed bank density [log] | 0.079 | 0.173 | <0.001 |  | max seed bank density [log] | ~~ | seed mass [log] | -0.089 | -0.228 | <0.001 |
|  |  |  |  |  |  |  |  | ~~ | seed dormancy (D) | 0.018 | 0.063 | 0.021 |

**Table S5** Structural equation models: Fit indices and significant indirect effects of mean or maximum seed bank density on the response variable, via seed bank type. Only significant indirect effects are reported, with both standardized and unstandardized path coefficients (in brackets) being presented. *n* = number of species. CFI = Comparative Fit Index, RMSEA = Root Mean Square Error of Approximation, SRMR = Standardized Root Mean Square Residual. Mean and maximum seed bank density (no. of seedlings m^-2^), seed mass (mg), and naturalization extent (no. of naturalized regions) are expressed on a log(*x*+1)-scale.

| **Model** | **Response** | **Response type** | **CFI** | **RMSEA** | **SRMR** | ***R^2^*** | ***n*** | **Significant direct effects** | **Significant indirect effects** |
| --- | --- | --- | --- | --- | --- | --- | --- | --- | --- |
| Model 1 | Naturalization incidence | binary (yes/no) | 0.999 | 0.019 | 0.008 | 0.064 | 2350 | seed bank type |  |
| Model 2 | Naturalization incidence | binary (yes/no) | 0.999 | 0.019 | 0.008 | 0.099 | 2350 | seed bank type  seed mass [log(x+1)] | seed bank type via max seed bank density [log(x+1)] 0.104 (0.103) |
| Model 3 | Naturalization extent | no. naturalized regions [log(x+1)] | 0.999 | 0.019 | 0.008 | 0.083 | 2350 | seed bank type  seed mass [log(x+1)]  seed dormancy | |
| Model 4 | Naturalization extent | no. naturalized regions [log(x+1)] | 0.999 | 0.019 | 0.008 | 0.140 | 2350 | seed bank type  seed mass [log(x+1)] seed dormancy | seed bank type via max seed bank density [log(x+1)]  0.128 (0.192) |
| Model 5 | Invasiveness | binary (yes/no) | 0.971 | 0.089 | 0.026 | 0.05 | 1253 | seed bank type |  |
| Model 6 | Invasiveness | binary (yes/no) | 0.986 | 0.089 | 0.027 | 0.058 | 1253 | seed bank type | seed bank type via max seed bank density [log(x+1)]  0.094 (0.096) |

**Figure S1**

**Fig. S1** Location of sites of origin of seed bank data collected for 2,350 flowering plant species from the native range, in 11,893 records (a) globally and (b) in Europe. (c) Number of seed bank records collected from seven geographic regions. Purple dots indicate sites supporting the seed bank of species that are classified as naturalized, while green dots indicate sites supporting only seed banks of species that are not classified as naturalized.

**Fig. S3** (a) Number of species in classes of naturalization incidence (naturalized vs non-naturalized) and (b) number of species in classes of naturalization extent (on a log(x+1)-scale), grouped by seed bank type (turquoise = persistent, salmon = transient).

**Figure S3**

**
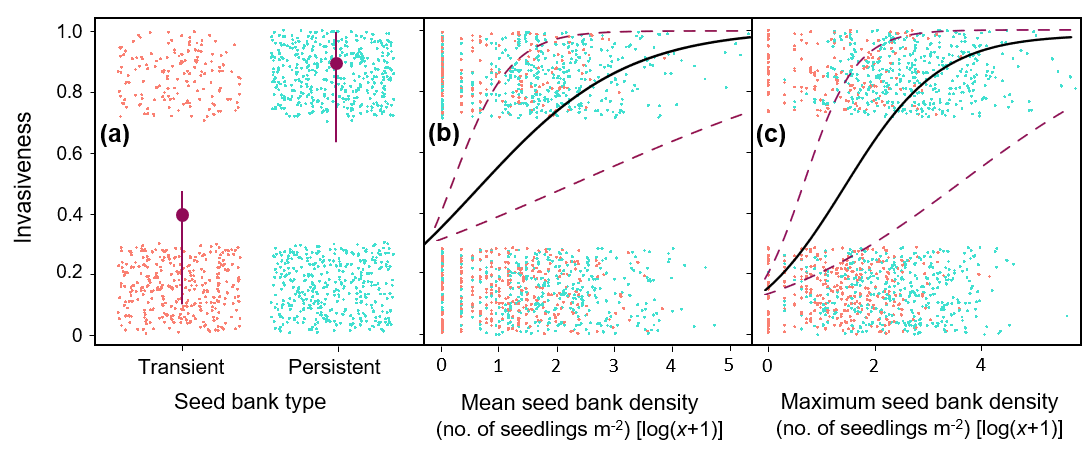
**

**Fig. S3** Probability of naturalized species becoming invasive in relation to seed bank properties and species traits. Fitted values from phylogenetically informed binary models of global invasion status (whether a naturalized species has become invasive somewhere in the world), in relation to (a) seed bank type (persistent vs. transient), (b) mean seed bank density (seedlings per square meter [log(*x*+1)]), and (c) maximum seed bank density (seedlings per square meter [log(*x*+1)]), for 1,253 naturalized flowering plant species, using MCMC sampling. Persistent seed banks are displayed in turquoise, while transient seed banks are presented in salmon. Jitter points were used to display all points within each level of the categorical variable invasiveness [invasive (1) vs non-invasive (0)].

**Figure S4**

**
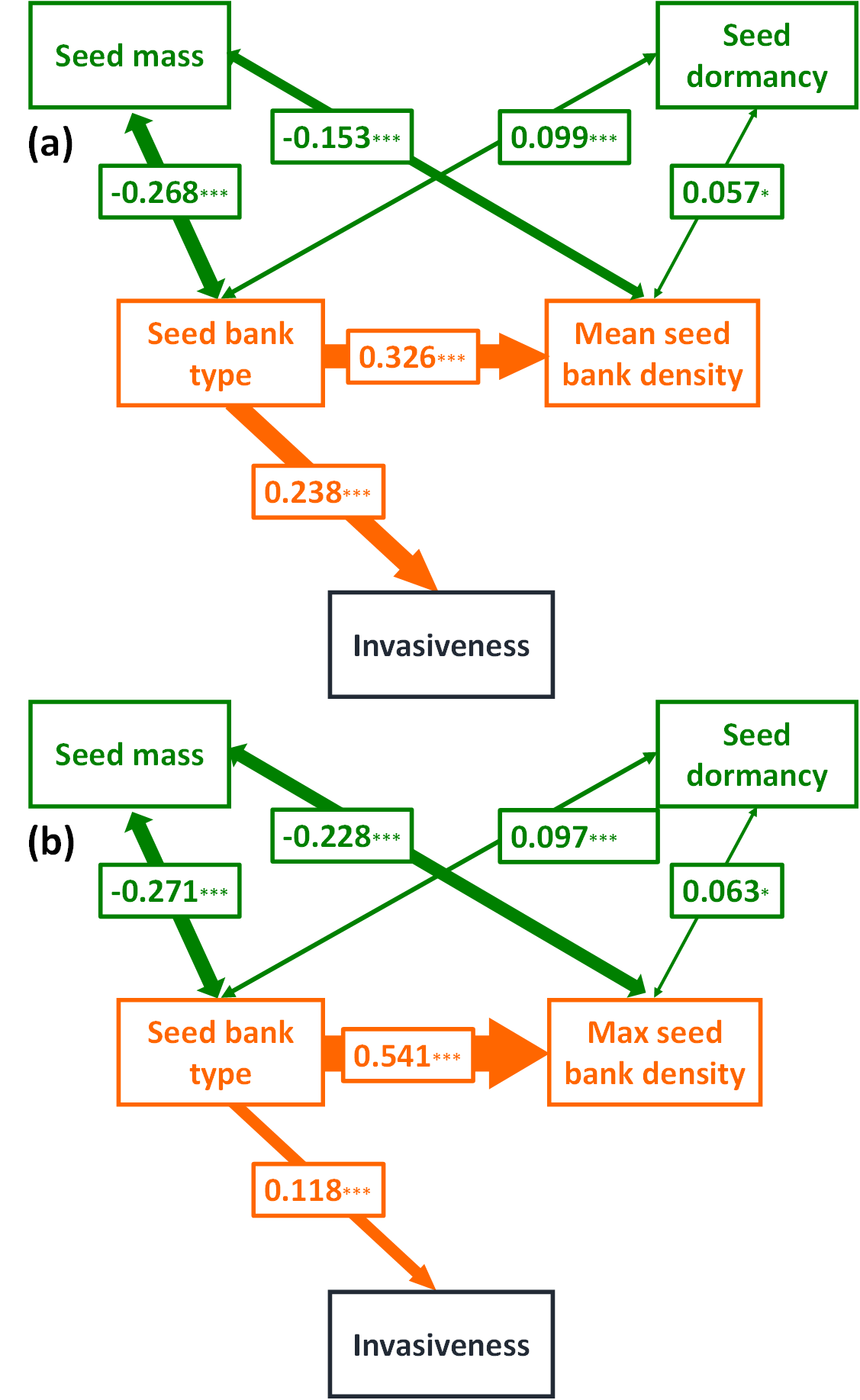
**

**Figure S4** Structural equation model considering all plausible pathways of direct and indirect effects on the global invasion status of naturalized species (Invasiveness), including direct effects of seed bank properties (type, mean density, maximum density) and seed traits (seed mass, seed dormancy) and indirect effect of seed bank type on Invasiveness through seed bank density (mean and max), for 1,253 naturalized flowering plant species. Seed bank density was included in terms of (a) mean density values and (b) maximum density values, at the species level. Only significant standardized coefficients are presented (**P* ≤ 0.05; ***P* ≤ 0.01; ****P* ≤ 0.001, *z*-test). The thickness of the solid and dashed arrows reflects the magnitude of the standardized SEM coefficients. Mean and maximum seed bank density (number of seedlings m^-2^), seed mass (mg), and naturalization extent (number of naturalized regions) are expressed on a log(*x*+1)-scale. Fit indices of the tested SEM models and *R*^2^ values are presented in Table S4.
